# MicroRNA dysregulation in ataxia telangiectasia

**DOI:** 10.3389/fimmu.2024.1444130

**Published:** 2024-08-19

**Authors:** Emilia Cirillo, Antonietta Tarallo, Elisabetta Toriello, Annamaria Carissimo, Giuliana Giardino, Antonio De Rosa, Carla Damiano, Annarosa Soresina, Raffaele Badolato, Rosa Maria Dellepiane, Lucia A. Baselli, Maria Carrabba, Giovanna Fabio, Patrizia Bertolini, Davide Montin, Francesca Conti, Roberta Romano, Elisa Pozzi, Giulio Ferrero, Roberta Roncarati, Manuela Ferracin, Alfredo Brusco, Giancarlo Parenti, Claudio Pignata

**Affiliations:** ^1^ Department of Translational Medical Sciences, Pediatric Section, Federico II University of Naples, Naples, Italy; ^2^ Istituto per le Applicazioni del Calcolo “Mauro Picone”, Naples, Italy; ^3^ Department of Clinical and Experimental Sciences, University of Brescia and Department of Pediatrics, ASST-Spedali Civili, Brescia, Italy; ^4^ Pediatric Area, Fondazione Istituto di Ricovero e Cura a Carattere Scientifico (IRCCS) Ca’ Granda Ospedale Maggiore Policlinico, Milan, Italy; ^5^ Department of Internal Medicine, Fondazione Istituto di Ricovero e Cura a Carattere Scientifico (IRCCS) Ca’ Granda Ospedale Maggiore Policlinico, Milan, Italy; ^6^ Unità Operativa Complessa (U.O.C) di Pediatria e Oncoematologia, Azienda Ospedaliero Universitaria Parma, Parma, Italy; ^7^ Department of Pediatric and Public Health Sciences, University of Torino, Torino, Italy; ^8^ Pediatric Unit, Istituto di Ricovero e Cura a Carattere Scientifico (IRCCS) Azienda Ospedaliero-Universitaria di Bologna, Bologna, Italy; ^9^ Centro Regionale di Biologia Molecolare – Arpa Piemonte, Torino, Italy; ^10^ Department of Clinical and Biological Sciences, University of Torino, Torino, Italy; ^11^ Istituto di Genetica Molecolare, Consiglio Nazionale delle Ricerche (CNR), Bologna, Italy; ^12^ Department of Medical and Surgical Sciences, University of Bologna, Bologna, Italy; ^13^ Department of Neurosciences Rita Levi Montalcini, University of Torino, Torino, Italy; ^14^ Unit of Medical Genetics, Città della Salute e della Scienza University Hospital, Torino, Italy

**Keywords:** ataxia telangiectasia, microRNA, immunodeficiency, cancer, DNA repair

## Abstract

**Introduction:**

Ataxia telangiectasia (AT) is a rare disorder characterized by neurodegeneration, combined immunodeficiency, a predisposition to malignancies, and high clinical variability. Profiling of microRNAs (miRNAs) may offer insights into the underlying mechanisms of complex rare human diseases, as miRNAs play a role in various biological functions including proliferation, differentiation, and DNA repair. In this study, we investigate the differential expression of miRNAs in samples from AT patients to identify miRNA patterns and analyze how these patterns are related to the disease.

**Methods:**

We enrolled 20 AT patients (mean age 17.7 ± 9.6 years old) and collected clinical and genetic data. We performed short non-coding RNA-seq analysis on peripheral blood mononuclear cells (PBMCs) and fibroblasts to compare the miRNA expression profile between AT patients and controls.

**Results:**

We observed 42 differentially expressed (DE)-miRNAs in blood samples and 26 in fibroblast samples. Among these, three DE-miRNAs, miR-342-3p, miR-30a-5p, and miR-195-5p, were further validated in additional AT samples, confirming their dysregulation.

**Discussion:**

We identified an AT-related miRNA signature in blood cells and fibroblast samples collected from a group of AT patients. We also predicted several dysregulated pathways, primarily related to cancer, immune system control, or inflammatory processes. The findings suggest that miRNAs may provide insights into the pathophysiology and tumorigenesis of AT and have the potential to serve as useful biomarkers in cancer research.

## 1 Introduction

Ataxia telangiectasia (AT) is a rare genetic disorder characterized by cerebellar neurodegeneration, telangiectasia, combined immunodeficiency, a predisposition to hematopoietic malignancy and cancer, and sensitivity to ionizing radiation (1, 2). At the biological level, alterations in cell cycle checkpoints, increased chromosomal breakage, and telomere end fusions have been well-documented and are considered to be involved in some phenotypic features of the disorder (3). AT is caused by mutations in the ataxia telangiectasia mutated (*ATM*) gene, which encodes a multifunctional serine/threonine protein kinase primarily involved in cell cycle regulation and repair of DNA double-strand breaks (4, 5). Despite various therapeutic approaches that have been explored, a cure for this genetic disorder is still unavailable, and only limited relief of neurologic symptoms can be achieved with supportive treatments (2). Recent studies have suggested that very low doses of glucocorticoids (GCs) may transiently improve the neurological symptoms and enhance lymphocyte proliferation in a small subset of AT patients even though these patients share the same genetic alterations (6–9).

The underlying pathophysiologic bases of phenotypic variability and of the variable response to treatments in AT have not been fully explored and remain unclear. In recent years, a comparative study using microarray analysis was conducted on AT patients who responded differently to GC treatment, revealing significant differences in gene expression profiles, particularly involving microRNAs (miRNAs) and multiple complex families. In particular, we observed that more than 600 miRNA-coding genes were upregulated in the responder patient, some of them being relevant in the pathogenesis of AT (10).

The investigation of miRNA expression profiles is a promising area of research in AT; miRNAs are short RNA molecules that play a role in post-transcriptional gene regulation by fine-tuning target mRNA expression. They belong to a family of non-coding RNAs, including long non-coding RNAs (lncRNAs), circular RNAs (circRNAs), ribosomal RNAs (rRNAs), small nuclear RNAs (snRNAs), small nucleolar RNAs (snoRNAs), and transfer RNAs (tRNAs). While the biological relevance of many of these molecules is still to be defined, miRNA profiling has the potential to offer insights into the pathophysiology of human diseases, as miRNAs are involved in various biological functions such as proliferation, differentiation, organogenesis, embryogenesis, and apoptosis (11–14). In addition, miRNAs are easily studied, not only in tissues but also in biological fluids, eliminating the need for invasive sampling procedures (15–17).

Furthermore, miRNAs are often located at fragile sites, as well as in regions of minimal loss of heterozygosity, amplifications (minimal amplicons), or common breakpoint regions (18). This has garnered increased attention as miRNAs could potentially be linked to chromosome-rearranged malignancies (19). Chromosome instability is a hallmark of AT, and at the cellular level, patients exhibit a high frequency of chromosomal rearrangements in various cell types compared to healthy individuals, with chromosomes 7 and 14 being the most commonly involved in T lymphocytes. Fibroblasts from AT patients also show a high rate of chromosomal rearrangements, typically not involving chromosomes 7 and 14 (20).

The objective of this study was to investigate the differential expression of miRNAs in blood cells from sporadic or familial AT patients and healthy individuals, aiming at identifying miRNA signatures associated with AT and exploring their correlation with the pathophysiology of the disease. Furthermore, the miRNA signatures in the blood were compared to those observed in fibroblasts to identify potential shared pathogenetic mechanisms contributing to the phenotypic expression in different tissues.

## 2 Materials and methods

### 2.1 Study design and participants

The study involved blood samples collected from 17 AT patients, followed up at six centers (Naples, Brescia, Milan, Bologna, Turin, and Parma) affiliated to the Italian Primary Immunodeficiency Network (IPINet). The initial diagnosis of AT was established based on clinical criteria and alpha-fetoprotein (AFP) values, in accordance with the guidelines of the European Society for Immunodeficiencies (ESID) and confirmed by *ATM* gene sequencing. AT patients who were undergoing treatment for lymphoproliferative disorder or other cancers, taking medications affecting extrapyramidal symptoms, or experiencing active infection were excluded from the study. Blood samples were collected during routine follow-up visits at the respective hospitals following standard procedures. Age distribution and gender distribution were similar between AT patients and the control group. Pediatric control samples were obtained from residual blood samples collected during routine tests in healthy cases undergoing medical screening for minor clinical conditions (such as uncomplicated asthma or short stature), who did not require additional medical interventions. Juvenile and adult control samples were obtained from healthy volunteers, who provided their blood for research purposes. Control participants were excluded if they exhibited signs or symptoms suggestive of immunological disorders, ataxia telangiectasia, or other neurodegenerative conditions or if they had used any medications in the past 3 months. Fibroblasts were obtained from three additional AT participants (ATF A–C; three male patients) and three healthy controls (two male patients).

### 2.2 Ethics and data protection

The study was approved by the Federico II Ethics Committee (protocol No. 66/18). Written informed consent was obtained from all participants or their parents/legal guardians. The study adhered to the ethical principles outlined in the Declaration of Helsinki and followed the guidelines of Good Clinical Practice.

### 2.3 Immunological evaluations

Data on lymphocyte counts, serum immunoglobulin levels (IgG, IgA, and IgM), and immunophenotype, including T cells (CD3^+^), helper T cells (CD3^+^CD4^+^), cytotoxic T cells (CD3^+^CD8^+^), naive T cells (CD4^+^ or CD8^+^, CD45RA^+^), B cells (CD3^−^CD19^+^), and natural killer (NK) cells (CD3^−^CD16^+^/CD56^+^), were collected and assessed using standard methods. These measurements were then compared to age-matched normal values for reference.

### 2.4 Sample collection and RNA extraction

Peripheral blood mononuclear cells (PBMCs) were isolated from heparinized blood by HiSep™ LSM 1077 (HiMedia, Modautal, Germany) density gradient centrifugation (21). For six patients, instead of PBMCs, we collected WBCs either with K3 Potassium EDTA anticoagulant agent or with PAXgene Blood RNA tubes (BD Biosciences, Franklin Lakes, New Jersey, United States, Cat. #762165), in order to preserve the quality of intracellular RNA (22). Fibroblast cell lines were obtained from skin biopsies and cultured in Dulbecco’s modified Eagle medium (DMEM, Sigma-Aldrich, Italy) with 2 mMol of glutamine, 50 U/ml of penicillin, 50 µg/ml of streptomycin, 1 mMol of sodium pyruvate, and 10% FBS. Total RNA, including small RNAs, was extracted from PBMCs using the miRNeasy Kit (Qiagen, Germantown, MD, USA) according to the manufacturer’s instructions. The quantity of RNA was measured using a spectrophotometer (NanoDrop 2000c; Thermo Scientific). The Agilent 2100 Bioanalyzer was used to assess the RNA integrity number (RIN) from PBMC samples. Those with RIN >7 were selected for library preparation. The quality of RNAs extracted from WBCs was assessed by gel electrophoresis on a 2% TBE-agarose gel. Total RNA was quantified using the Qubit 4 fluorometric assay (Life Technologies/Thermo Fisher Scientific, MA, USA).

### 2.5 Next-generation sequencing library preparation and data alignment

#### 2.5.1 Blood cells

Libraries were prepared from 250 ng of total RNA using the Negedia small RNA-seq sequencing service provided by Next Generation Diagnostics srl. This service included library preparation, quality assessment, and sequencing on a NovaSeq 6000 sequencing system using a single-end, 50-cycle strategy (Illumina, Cambridge, UK). The raw reads were trimmed to remove adapter sequences and low-quality ends using Trim Galore (v0.6.2). The filtered reads of the samples were aligned to human mature miRNAs (miRBase release 21) using Bowtie (v1.2.2). Raw counts were determined with SAMtools (v1.9).

#### 2.5.2 Fibroblasts

Small RNA libraries were created for six fibroblast samples using TruSeq Small RNA Library PrepKit v2 (Illumina, San Diego, CA RS-200-0012/24/36/48). The libraries were generated following the manufacturer’s instructions starting from 35 ng of purified RNA. The High Sensitivity DNA (HS-DNA) kit (Agilent Technologies, Santa Clara, CA, USA 5067-4626) was used to quantify the libraries using Agilent Bioanalyzer (Agilent Technologies). The 24 DNA libraries were combined in equal amounts to create a library pool. The pooled libraries underwent size selection using magnetic beads (Agencourt, Beckman Coulter, Brea, CA). They were then quantified using the HS-DNA Kit (Agilent Technologies), denatured, neutralized, and combined with a PhiX control library (standard library normalization). A final concentration of 1.8 pM of the pooled libraries was obtained and sequenced using the NextSeq 500/550 High Output Kit v2 (75 cycles) (Illumina, FC-404-2005) on the Illumina NextSeq500 platform. The raw base-call data were demultiplexed using Illumina BaseSpace Sequence Hub and converted to FASTQ format. After a quality check with the FastQC tool, adapter sequences were trimmed using Cutadapt, which also removed sequences shorter than 16 nucleotides and longer than 30 nucleotides. Reads were mapped using the STAR algorithm (23). Only reads that mapped unambiguously to the reference genome retrieved from the miRBase 21 database (with at least 16 nucleotides aligned and allowing a 10% mismatch) were used for the downstream analyses. Raw counts from mapped reads were obtained using the htseq-count script from the HTSeq tools (24).

### 2.6 Bioinformatics analysis

The differential expression miRNA analysis for both PBMC and fibroblast samples was performed using DESeq2 (version 1.38.3), a statistical package based on generalized linear models suitable for multifactorial experiments. KEGG pathway enrichment analysis of miRNA targets was performed using the miRNet tool, a network-based visual analytics for microRNA functional analysis and systems biology, with an FDR threshold of <0.05 for significant enrichment. Graphical representations were created using the SR-PLOT and RawGraphs tools (25). Reactome and WikiPathways analysis based on the microRNA signature was performed using the web tool MIENTURNET (MicroRNA ENrichment TURned NETwork) for miRNA-195-5p, miRNA-30a-5p, and miRNA-342-3p.

### 2.7 Quantitative real-time polymerase chain reaction

To obtain cDNA, reverse transcription was carried out using either the TaqMan Advanced miRNA cDNA Synthesis Kit or the TaqMan miRNA Reverse Transcription kit (Applied Biosystems, Foster City, CA, USA). The expression of mature miRNAs was assessed using TaqMan MicroRNA Assay (Applied Biosystems, Foster City, CA) for the miRNA selected for validation (hsa-miR-342-3p assay ID 478043_mir, hsa-miR-195-5p assay ID 477957_mir, hsa-miR-30a-5p assay ID 000417_mir). Quantitative real-time polymerase chain reaction (qRT-PCR) was performed on a Roche LightCycler® 480 System (Roche Diagnostics, Risch-Rotkreuz, Switzerland) using a TaqMan Fast Advanced Master Mix (Cat. #4444557). Samples were run in duplicate, and single TaqMan microRNA assays were performed according to the manufacturer’s instructions (Applied Biosystems, Foster City, CA). Differences in miRNA expression, presented as fold changes, were calculated using the 2^−ΔΔCt^ method; miR-93 (assay ID 478210_mir) was used as an endogenous normalizer in tissue samples. Each analysis was repeated in three independent experiments.

### 2.8 Statistical analysis

Statistical analysis was performed using GraphPad Prism version 9.1 software with the Student’s *t*-test method. Data were deemed statistically significant with *P* ≤0.05 (*), *P* ≤0.01 (**), or *P* ≤0.001 (***).

## 3 Results

### 3.1 Demographic and genomic features of AT patients

**Table 1** summarizes the demographic and the main clinical phenotypic features of AT patients at the time of enrollment. Except for three patients, all were of Italian origin. The mean age ± SD at disease onset was 26.6 + 22.8 months (median 20), the mean age at diagnosis was 7.3 ± 7.7 years (median 4.5, data not shown), while the mean age at enrollment was 17.7 ± 9.6 (median 16.5, range 6–43) years. The cohort consisted of 4 patients <10 years of age, 10 patients aged 11–20 years, and 6 patients over 21 years of age, representing the different stages of the disease. Pairs AT1 and AT6, AT4 and AT17, and AT9 and AT10 were siblings. Most patients were compound heterozygotes (14/20), while six had homozygous mutations (**Supplementary Table 1**). Frameshift mutations were the most common. Patients AT1 and AT6 were identified to have very low ATM kinase activity due to being homozygous for the c.3576G>A mutation (26, 27).

**Table 1:** Demographic and clinical characteristics of AT patients.

Patient ID	Sex	Ethnicity	AO (months)	AE (years)	SARA	Wheelchair confined	Infections/Lung disease	Immunodeficiency	Autoimmunity	Cancer
AT1	F	Non-Hispanic	72	41	25	Yes	No	HIGM	AIT	Gastric cancer
AT2	M	Other	26	14	22.5	Yes	No	Low naive T cells, defective proliferative response	Antinuclear antibodies +	No
AT3	M	Non-Hispanic	12	21	NA	Yes	Yes	Pan-hypogammaglobulinemia, low CD19^+^	No	ALL
AT4	F	North-African	23	10	15.5	No	No	sIgAD	Rheumatic fever	No
AT5	F	Non-Hispanic	60	11	NA	No	Yes	Low IgG, IgA, naive T cells, defective proliferative response	No	No
AT6	F	Non-Hispanic	72	43	32	No	No	HIGM, low IgG, IgA	AIT	No
AT7	F	Non-Hispanic	24	21	24	Yes	Yes	Low CD3^+^, CD4^+^, CD8^+^, HIGM	No	No
AT8	F	Non-Hispanic	72	11	16	No	Yes	Low naive T cells, defective proliferative response	No	No
AT9	F	Non-Hispanic	12	8	NA	No	Yes	Low IgG, IgA, CD3^+^, CD4^+^, CD8^+^ T cells, defective proliferative response	No	Burkitt lymphoma
AT10	F	Non-Hispanic	12	6	NA	No	Yes	Low IgG, IgA, CD3^+^, CD4^+^ T cells, defective proliferative response	No	No
AT11	F	Non-Hispanic	18	24	22	Yes	No	Low CD3^+^, CD4^+^, CD8^+^, naive T cells, IgA, defective proliferative response	No	No
AT12	M	Non-Hispanic	19	18	24	Yes	Yes	Low CD3^+^, CD4^+^, CD8^+^, naive T cells, IgA, defective proliferative response	AIT	No
AT13	F	Non-Hispanic	7	9	16	No	No	sIgAD	No	No
AT14	M	Non-Hispanic	1	20	22	Yes	No	Low CD4^+^, CD8^+^ naive T cells, defective proliferative response	No	No
AT15	F	Non-Hispanic	21	12	NA	No	Yes	Low CD8^+^, low IgA	No	No
AT16	M	Non-Hispanic	9	18	NA	No	Yes	Low CD3^+^, CD4^+^, CD8^+^, IgG, HIGM	Psoriasis	No
AT17	M	North-African	12	15	16	No	No	Low IgG, IgA	No	No
**ATF A**	M	Non-Hispanic	24	15	NA	Yes	No	Low CD4^+^, pan-hypogammaglobulinemia	No	No
**ATF B**	M	Non-Hispanic	12	18	NA	Yes	Yes	Low CD8^+^	No	No
**ATF C**	M	Non-Hispanic	24	18	NA	Yes	No	No	No	No

AO, age at onset; AE, age at enrollment; SARA, Scale for Assessment and Rating of Ataxia; HIGM, high IgM; AIT, autoimmune thyroiditis; ALL, acute lymphoblastic leukemia; sIgAD, selective IgA deficiency; NA, not available.

### 3.2 Clinical features

All subjects had gait ataxia, 18/20 had oculomotor apraxia, and 10/20 were wheelchair-confined. Additional neurological manifestations included muscle hypotonia in 5/20 patients, chorea and dystonia in 4/20 patients, and intellectual disability or developmental delay in 4/20. When available, data about neurological assessment using the Scale for the Assessment and Rating of Ataxia (SARA) are reported in **Table 1**. In this study, the average SARA score of AT patients was 21.9 ± 5.3, ranging from 15.5 to 32, thus including patients with maximal or total dependence in performing daily living activities (SARA maximal impairment score is 40).

The most common reported infections were recurrent sinopulmonary infections (6/20, 30%), interstitial lung disease (2/20, 10%), bronchiectasis (3/20, 15%), recurrent otitis (5/20, 25%), bronchiolitis (2/20, 10%), sepsis (1/20, 5%), septic arthritis (1/20, 5%), and VZV recurrent infections (1/20, 5%). None of the patients had active infections at the time of enrollment. Seven of them were on antibiotic prophylaxis with cotrimoxazole (AT2–4, AT9–10, AT16–17), one patient was on penicillin prophylaxis for a previous diagnosis of rheumatic disease (AT4), and four were receiving immunoglobulin replacement therapy (AT3, AT9, AT17, and ATF A). Two patients were taking vitamins C and D (AT2 and AT14). Regarding immune dysregulation, 5/20 (25%) patients showed autoimmune disorders, particularly autoimmune thyroiditis (3/20, 15%) and psoriasis (1/20, 5%), while one subject had persistent humoral signs of autoimmunity. Overall malignancies were reported in three patients. AT9 had a previous diagnosis of Burkitt lymphoma, AT3 was diagnosed as affected with acute lymphoblastic leukemia after a few days from the enrollment, and AT1 developed gastric cancer during follow-up.

### 3.3 miRNA profile in PBMCs and fibroblasts in AT patients

We performed small non-coding RNA-seq analysis on AT PBMCs and fibroblasts to identify specific expression signatures for AT. Initially, this analysis was carried out on PBMC samples from the first 10 AT patients referred to our unit and on fibroblasts from an additional three patients. The results from AT patients were compared to those obtained in respective age-matched healthy control samples (PBMCs from eight patients and fibroblasts from three controls). The flowchart outlining this process is shown in **Supplementary Figure 1** (in this article’s Online Repository).

**Figure 1:**
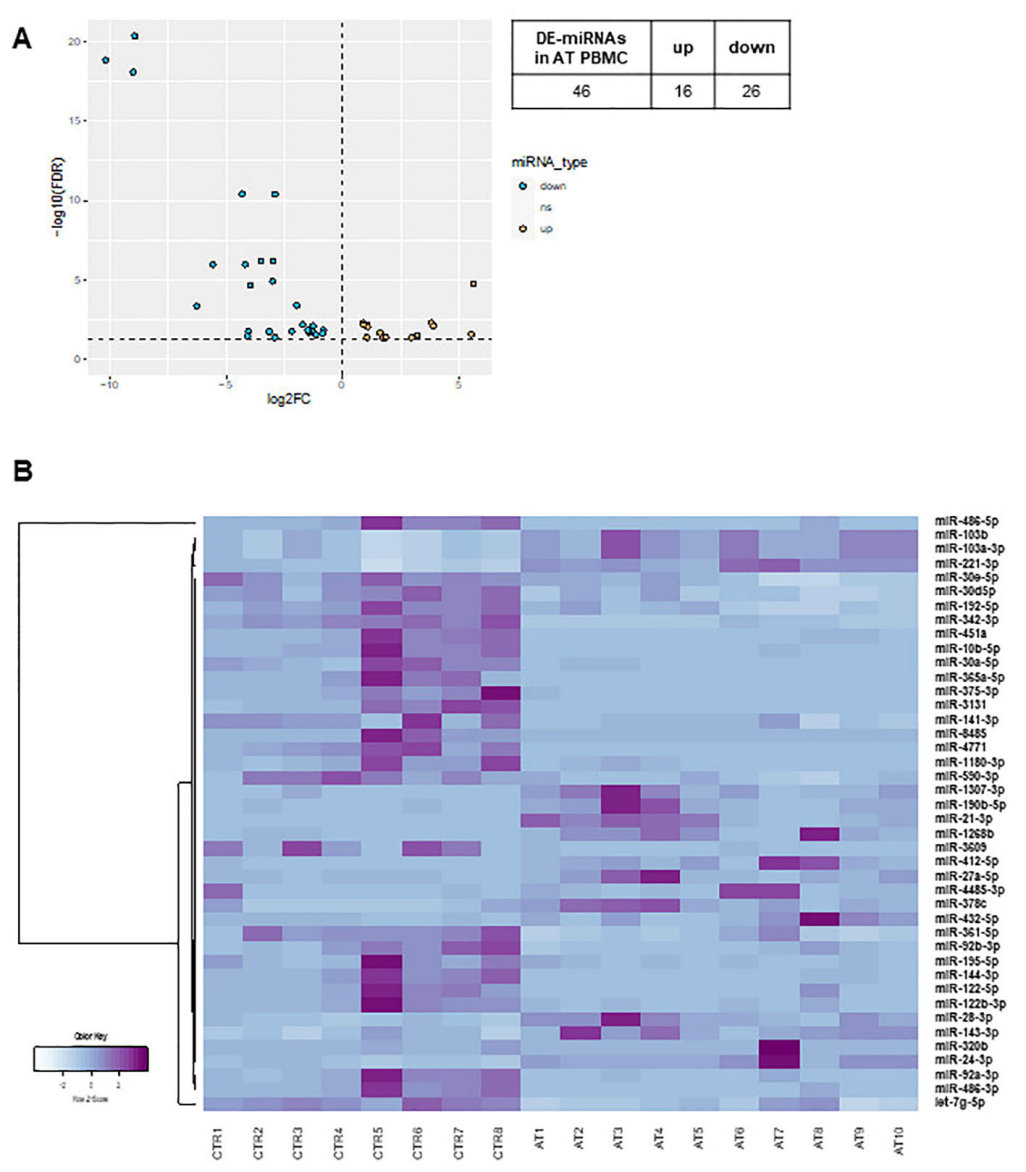
DE-miRNA in AT PBMCs. The volcano plot of DE-miRNAs **(A)**. Hierarchical clustering analysis of DE-miRNAs **(B)**. DE-miRNAs, differentially expressed miRNAs; FDR, false discovery rate; FC, fold change. Light blue: low expression, purple: high expression.

**Figure 2:**
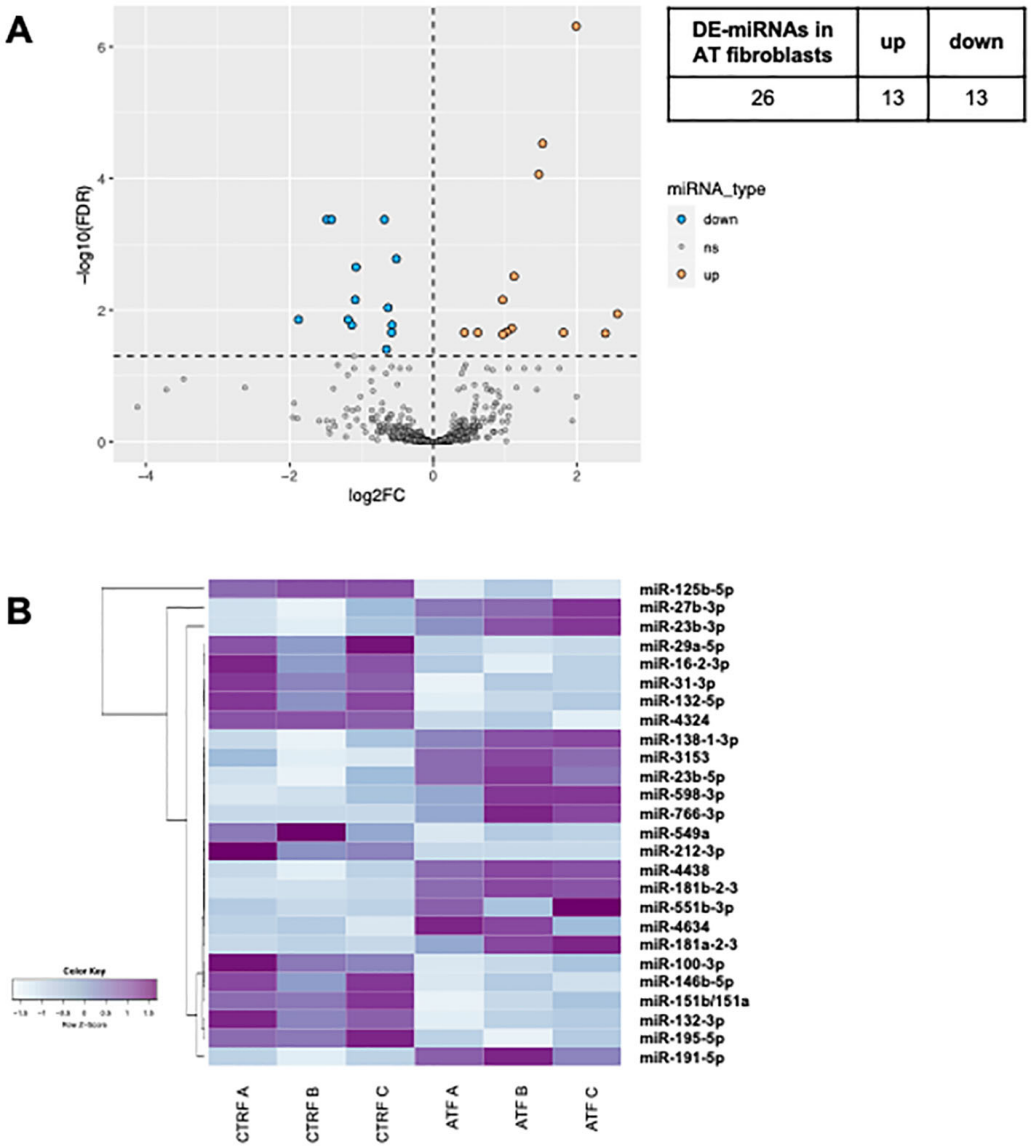
DE-miRNAs in AT fibroblasts. The volcano plot of DE-miRNAs **(A)**. Hierarchical clustering analysis of DE-miRNAs **(B)**. DE-miRNAs, differentially expressed miRNAs; FDR, false discovery rate; FC, fold change. Light blue: low expression, purple: high expression.

**Figure 3:**
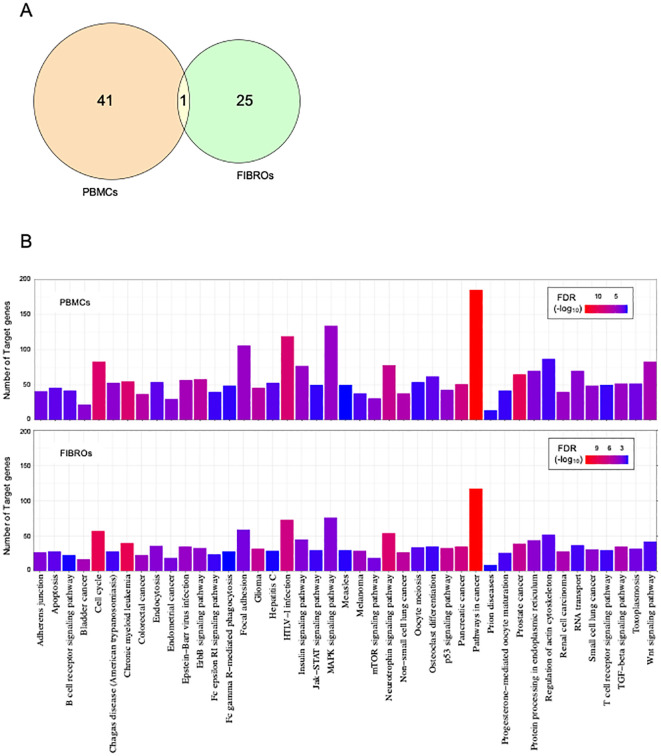
Cross-analysis of AT PBMCs and fibroblasts. Venn diagram (or map) of intersection DE-miRNAs in AT **(A)**. Indicated in orange are PBMCs DE-miRNAs; indicated in green are fibroblast DE-miRNAs; indicated in yellow are common DE-miRNAs. Enrichment analysis for target genes of DE-miRNAs in PBMCs at the top and in fibroblasts at the bottom **(B)**. Significant KEGG pathways with FDR ¾0.05. The significance increases from blue to red.

In both AT PBMCs (**Figure 1**) and fibroblasts (**Figure 2**), miRNA expression profiles differed from those found in their respective controls. In PBMCs, the analysis identified 42 differentially expressed miRNAs in AT patients compared to controls (DE-miRNAs) with statistical significance (FDR < 0.05) (**Figure 1A**). These DE-miRNAs were further classified into up- (16 miRNAs) or downregulated (26 miRNAs) in AT patients (**Figures 1A, B**). In fibroblasts from AT patients, 26 DE-miRNAs were identified, with 13 miRNAs showing upregulation and 13 showing downregulation (**Figures 2A, B**). Notably, miR-195-5p, a DE-miRNA not previously linked to AT, was found to be downregulated both in PBMCs and fibroblasts, suggesting a potential involvement in shared disease-relevant pathways despite differences in sample source and cell lineage commitment (**Figure 3A**).

Literature analysis indicates that dysregulated miRNAs regulate genes involved in various functions and pathways, particularly those related to cancer. To explore the functional roles of the differentially expressed miRNAs identified in our analyses, we conducted a KEGG pathway enrichment analysis using the miRNet tool. Specifically, we used the 42 DE-miRNAs identified in AT PBMCs and the 26 DE-miRNAs identified in AT fibroblasts as independent queries for pathway enrichment analysis.

We identified a shared set of dysregulated pathways involving the target genes of DE-miRNAs (**Figure 3B**). Most of these pathways were found to be associated with cancer-related processes. However, bioinformatic analysis uncovered additional pathways relevant to the pathophysiology of AT, such as neurotrophin signaling pathways, cell cycle regulation, TGF-beta signaling pathways, P53 signaling, mTOR signaling, apoptosis pathways, and B- and T-cell receptor pathways.

### 3.4 DE-miRNA validation by real-time PCR

Subsequently, we selected three DE-miRNAs (miR-195-5p, miR-342-3p, and miR-30a-5p) for further validation through qRT-PCR in an expanded cohort of AT patients. This validation included three of the patients previously analyzed by next-generation sequencing (NGS), along with seven additional patients (AT11–17) (summarized in **Supplementary Figure 2A**). The choice of these three miRNAs was based on specific criteria: miR-195-5p due to its consistent downregulation in both AT PBMCs and fibroblasts and miR-342-3p, recognized as a tumor suppressor miRNA, with previous associations to neural development (28–30), lymphoid and myeloid differentiation (31), and documented downregulated in various cancer types, including acute myeloid leukemia, breast cancer, and colon carcinoma. Additionally, miR-342-3p has also been implicated in neurodegenerative diseases such as amyotrophic lateral sclerosis (32). Lastly, miR-30a-5p was chosen for its reported role in inhibiting cancer cell proliferation, regulating cell cycle state, and promoting apoptosis. In particular, miR-30a has been shown to be involved in modulating the radiosensitivity effects in ATM cells (33).

**Figure 4:**
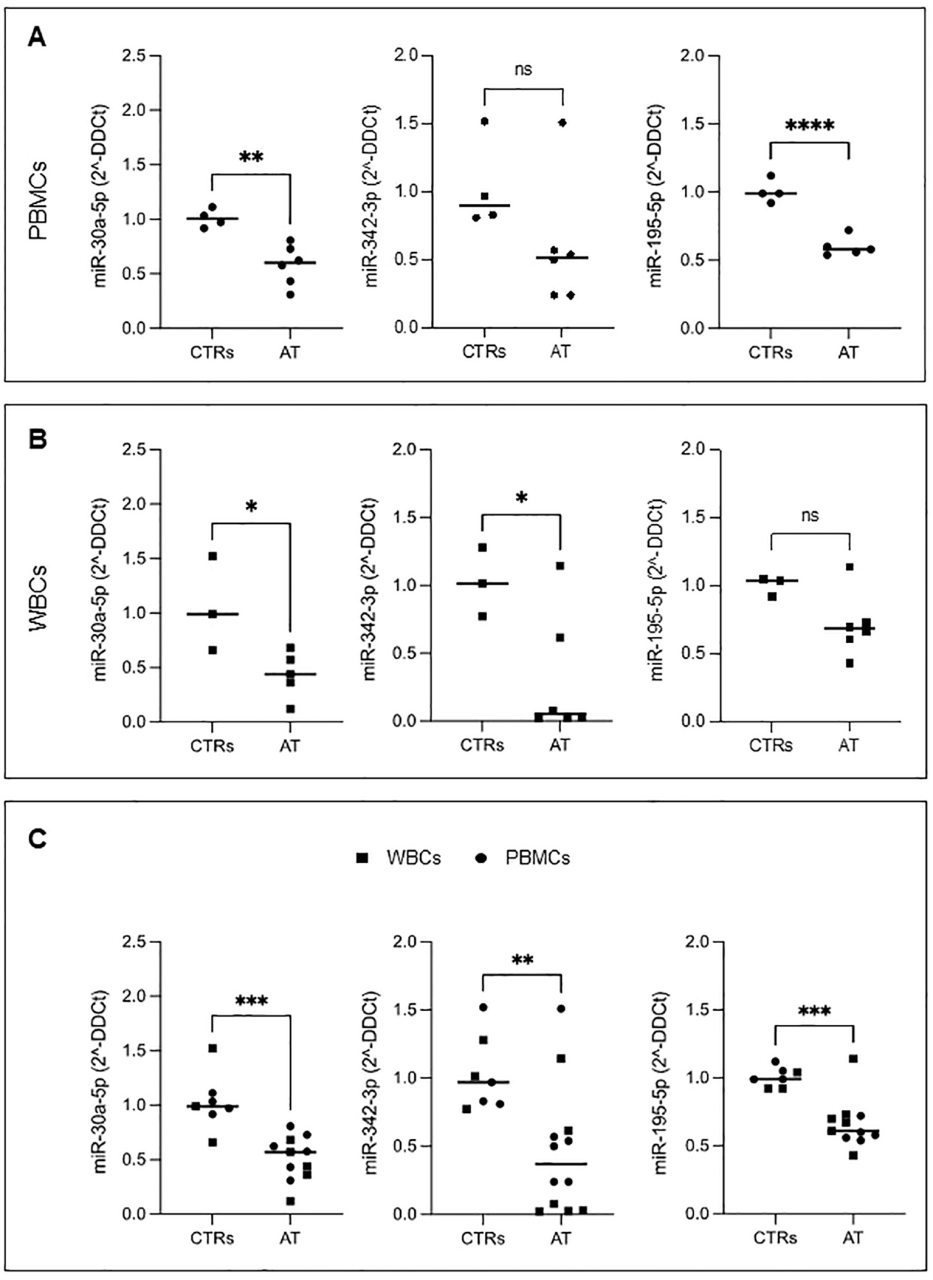
The expression level of three selected DE-miRNAs. Real-time qPCR of miR-30a-5p, miR-342-3p, and miR-195-5p in PBMCs **(A)** and WBCs **(B)**. The entire cohort of patients analyzed (union of PBMCs and WBCs) **(C)**. Student’s *t*-test was performed; ns, not significant, *P ¾ 0.05, **P ¾ 0.01, ***P ¾ 0.001.

In all AT PBMC samples, we confirmed the downregulation of miR-195-5p and miR-30a-5p (**Figure 4A**), whereas the downregulation of miR-342-3p, although following a similar trend, did not reach statistical significance.

We performed qRT-PCR analysis on total WBC samples from five patients (see **Supplementary Figure 2A**). In WBCs, miR-30a-5p and miR-342-3p were found to be significantly downregulated, in contrast to miR-195-5p (**Figure 4B**).

The overall results from the entire patient cohort, encompassing both PBMCs and WBCs, show similar patterns that align with the data obtained from NGS analysis. This consistency is further supported by the findings in patients AT11 and AT12 (where both sample types were available), consistently demonstrating the downregulation of miR-195-5p, miR-342-3p, and miR-30a-5p. These results indicate that the findings from WBCs are coherent with those from PBMCs (**Supplementary Figure 2B**).

Given the consistency of results in PBMCs and WBCs, we performed a statistical analysis of the combined results from both sample types. In this analysis, all the miRNAs studied were found to be significantly downregulated compared to controls (**Figure 4C**).

### 3.5 *In silico* analysis of interacting pathways of selected DE-miRNAs

**Figure 5:**
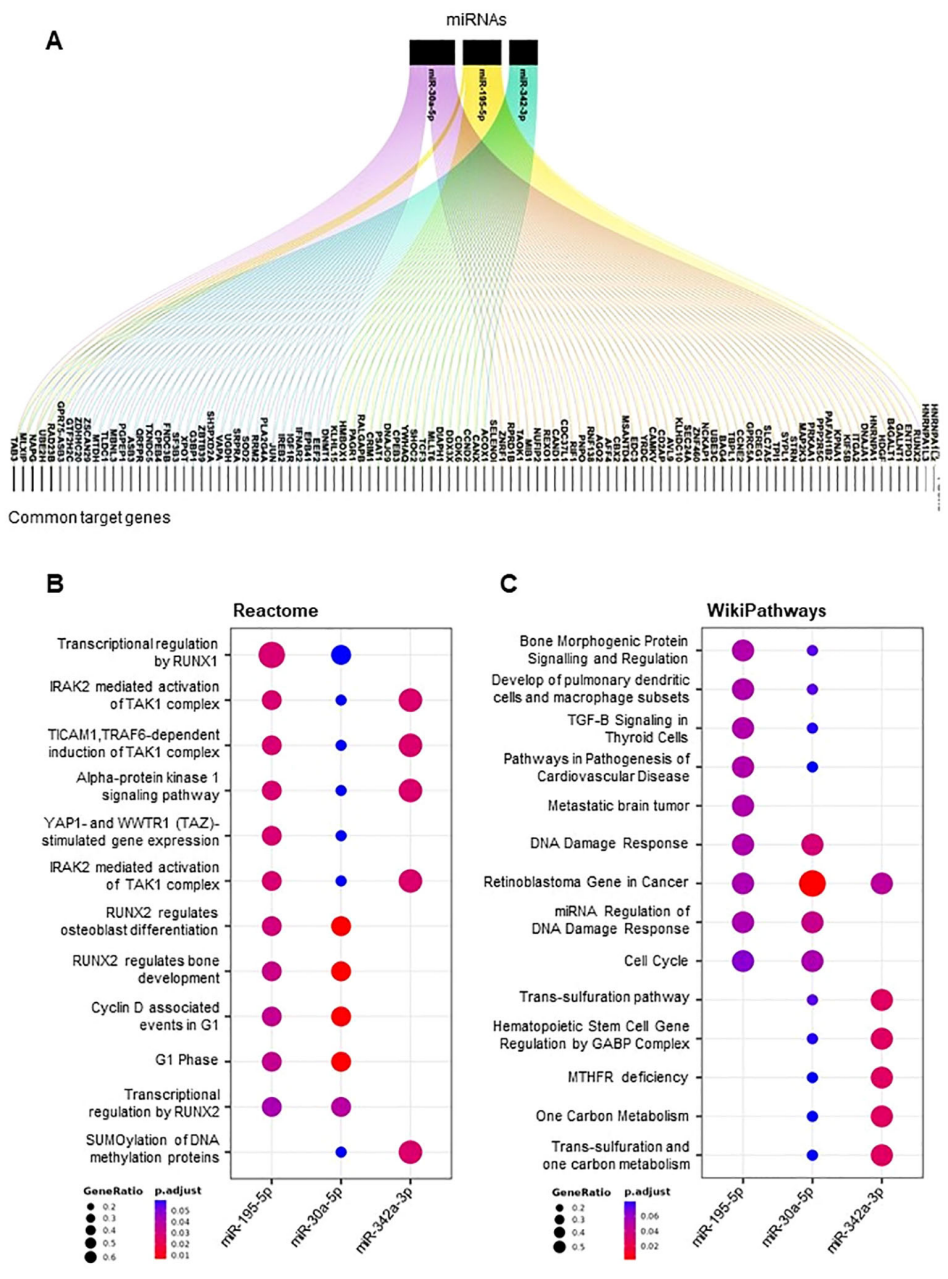
Target genes and pathways of selected DE-miRNAs. Alluvial plot showing a list of miR-30a-5p, miR-342-3p, and miR-195-5p common target genes **(A)**. Dot plot of functional enrichment analysis of all target genes of miR-30a-5p, miR-342-3p, and mi-195-5p. Pathway analyses by Reactome **(B)** and Wikipathways **(C)**. Significant pathways are listed and represented by circles colored according to the significance of the enrichment, and their size is proportional to the number of target genes regulating the described signaling pathways.

To delve deeper into the biological functions of miR-195-5p, miR-342-3p, and miR-30a-5p, we performed additional bioinformatic investigations focused on these three miRNAs. We retrieved a set of predicted target genes for each miRNA and determined the common target genes among them (**Figure 5A**). Several genes were identified as common targets of at least two of the miRNAs, and five genes (*TAB3*, *MLXIP*, *NAPG*, *UBE2H*, and *RAD23B*) emerged as shared targets of all three miRNAs.

Using the Reactome pathways and WikiPathways, we found that these common target genes converge into common pathways which include RUNX1- and RUNX2-related pathways, mechanisms related to p53 activation by DNA damage, immune response associated with the TAK1–TAB3 complex, and DNA damage response pathways (**Figures 5B, C**).

## 4 Discussion

In the current study, we identified a miRNA signature in blood cells and fibroblast samples obtained from a cohort of AT patients, whose phenotype was representative of the different stages of disease.

AT is the prototype of DNA repair disorders, characterized by progressive cerebellar neurodegeneration, combined immunodeficiency, chromosome instability, and susceptibility to hematological and solid cancer due to defect in *ATM* gene, a serine/threonine protein kinase which acts mainly through the phosphorylation of several tumor suppressor proteins. Malignancies, particularly lymphoma and leukemia in childhood and breast and gastric cancer in adulthood, together with lung disease represent the main causes of death in AT patients, which generally occurs by 25–30 years of life. Several studies have offered insights into the roles of miRNAs in neurodegenerative diseases and cancer, including pediatric hematological disorders and solid tumors (34). It has been documented that miRNAs can regulate DNA repair protein-coding genes and molecules associated with cell cycle checkpoints (35, 36). Liu et al. reported an ATM-dependent nuclear export of pre-miRNAs after DNA damage, suggesting a potential role of miRNAs in AT pathogenesis (37).

Research specifically investigating the dysregulation of miRNAs in AT patients is currently scarce. In a prior study, we highlighted a significant disparity in the expression levels of differentially expressed genes among AT patients who responded versus those who did not respond to treatment with GCs, with a majority of these genes being attributed to miRNA genes (10).

In this study, we performed an miRNA profiling on PBMCs and fibroblast samples from AT patients. We identified a set of 42 DE-miRNAs in PBMCs and 26 DE-miRNAs in fibroblast samples. After performing a cross-analysis, we identified several pathways that were consistently predicted to be deregulated in both AT PBMCs and fibroblasts. These pathways were found to be aligned with the AT pathophysiology and phenotype. Most were relevant to cancer-related processes (Wnt signaling pathway, MAPK signaling pathway, TGF-β signaling pathway), while others were associated with immune system regulation, inflammatory processes (such as T- and B-cell receptor signaling, JAK–STAT signaling pathway, apoptosis, or Fc receptor-mediated signaling), endocrine process (insulin signaling and oocyte maturation/meiosis), and neurological pathways (neurotrophin and TGF beta signaling pathway, Wnt and mTOR).

Among pathways relevant to AT, those involving the immune response to infectious agents such as HTLV1 and EBV were of particular interest, because miRNAs play a role not only in controlling and eliminating transcripts from DNA elements of the genome but also in the degradation of exogenous RNA, including that of viral origin. This suggests a potential role of intracellular miRNA in controlling infections, particularly those caused by oncogenic viruses (38–40). Another pathway of interest is related to the regulation of the actin cytoskeleton. In recent years, it has been reported that ATM interacts with the cytoskeleton, phosphorylating several cytoskeleton proteins and chromatin, thereby promoting cell survival during interstitial migration (41).

Among the three miRNAs we selected for further validation, miR-342-3p (previously known as miR-342) stood out as particularly promising. It is an intronic miRNA of the Enah/Vasp-like (EVL) host gene, located on chromosome 14q32, and is known to be involved in cancer as a tumor suppressor and kinase regulator. In mice, a significant and stable decrease in miR-342-3p has been observed after total body irradiation with X-rays, making it a potential biomarker for radiation damage. Moreover, increasing its levels with synthetic mir-342-mimetics has been shown to mitigate the radiation-induced depletion of lymphocytes, suggesting a role for its downregulation in T- and B-cell lymphopenia observed in AT patients (42).

Chromosome instability, reflected by an elevated level of spontaneous chromosome breakage in peripheral lymphocytes and fibroblasts, is a hallmark of AT. In particular, translocation in chromosomes 7 and 14, involving the T-cell receptor genes located at 14q32, is a common cytogenetic alteration observed in T lymphocytes of AT patients, with approximately a 40-fold increase in AT patients compared to healthy control (43). Furthermore, a high prevalence of chromothripsis, a catastrophic genomic event characterized by massive DNA rearrangements on one or few chromosome, has been reported in AT patients with tumors, mainly involving the acrocentric chromosome 14 (44).

Translocation of 14q32 has been shown to be associated with loss of heterozygosity and potential deletion of tumor suppressor gene loci. Chromosome loss inherent with 14q32 translocation may involve or inactivate miR-342-3p. miR-342-3p has also been identified as a differentially expressed miRNA, primarily downregulated, in various tumor cells including acute and chronic leukemia, hepatocellular carcinoma, glioma, and ovarian cancer. Its expression levels are closely correlated with disease staging, metastasis, and prognosis (32, 45–47).

The miR-30 family is a complex family with significant roles in humans [miR-30a, miR-30b, miR-30c (miR-30-c1 and miR-30-c2), miR-30d, and miR-30e]. Among them, miR-30a is recognized as a potential tumor suppressor involved in regulating cellular proliferation, apoptosis, migration, and invasion of diverse tumor cells; miR-30a-5p is involved in neuronal damage and is upregulated in models induced by amyloid beta-peptide (Aβ) (48).

In a recent study, plasma levels of mir30a-5p were significantly lower in patients with gastric cancer, showing a correlation with the presence of distant metastases, advanced stage of the disease, and tumor differentiation. Gastric cancer ranks as the fourth common cancer worldwide and the second leading cause of cancer-related mortality. It has been reported in both AT patients and AT carriers (49, 50). Despite advancement in survival rates due to improved endoscopic and imaging procedures, as well as medical and surgical services, its prognosis continues to be poor especially in patients with inborn errors of immunity (51).

miR-195-5p, a derivative of miR-195 precursors from the 5p arms, is known to influence genes that primarily regulate cell proliferation and apoptosis. It has been observed to correlate with various types of human solid cancers, including digestive and genitourinary malignancies, and has known inhibitory effects on the growth of leukemia cells (52). Furthermore, miR-195 is involved in modulating two of the proinflammatory cytokines, IL-6 and IL-8, whose dysregulation has been documented in AT patients. Elevated serum IL-8 levels in AT patients have been associated with a higher risk of malignancy and mortality (53).

Using bioinformatics analyses, we identified several genes targeted by the three validated miRNAs. Interestingly, five of them, *TAB3*, *MLXIP*, *NAPG*, *UBE2H*, and *RAD23B*, are primarily involved in malignancies, DNA damage response, cell cycle, and immune system and inflammatory response.

Evidence-based standards for cancer screening do not currently exist for AT patients, especially in childhood. AT patients often have elevated serum levels of alpha fetoprotein (AFP) (54), which is a biochemical marker of AT along with chromosomal instability (55). However, AFP is not predictive of malignancies in AT patients (56). The commonly used biomarkers for screening and monitoring gastric cancer, such as carcinoembryonic antigen (CEA) or carbohydrate antigens CA19-9 and CA72-4, have limited sensitivity and specificity and are not effective in detecting premalignant lesions. Therefore, there is a need to discover new biomarkers.

In our patient cohort, we observed a significant reduction in the levels of miR-342-3p, miR-30a-5p, and miR-195-5p in both PBMCs and WBCs. Among the patients, three (15% of the cohort) had malignancies. This suggests a potential role for these miRNAs in chromosomal instability and tumorigenesis, indicating their potential use as early biomarkers of malignancy in AT patients. However, further validation is needed through a prospective evaluation of a larger group of AT patients.

### 4.1 Study limitations

The limitations of our study are primarily attributed to the small sample size utilized. The identified miRNAs should be validated in a larger cohort of AT patients of different ages and clinical phenotypes to confirm the findings. In particular, the observation reported on fibroblasts should be implemented with additional samples to infer higher biologic significance. Further validation studies are warranted at the mRNA and protein levels to elucidate the association between the miRNAs identified and their target genes.

## Data Availability

The datasets presented in this study can be found in online repositories. The names of the repository/repositories and accession number(s) can be found below: https://www.ncbi.nlm.nih.gov/geo/, GSE266411 and https://www.ncbi.nlm.nih.gov/geo/, GSE266440.

## Supplementary material

Supplementary Figure 1 Schematic workflow of the next generation sequencing analyses. The analysis was performed in PBMCs *(orange)* and fibroblasts *(green)* from AT patients and controls.

Supplementary Figure 2 AT blood cells and fibroblasts samples table. Patients were analyzed through NGS, qRT-PCR or both. Blood cells from AT patients were PBMCs, WBCs or both. NGS was performed on PBMC from AT1- AT10 patients and on fibroblasts of ATF A-C patients. qRT-PCR was performed on WBC (AT3), PBMC (AT5 and AT7) and on further 7 AT patients. Among these, the analysis was performed on both WBC and PBMC (AT1 and AT12), PBMC (AT13 and AT14) or WBC (AT15-AT17) **(A)**. qRT-PCR of miR-30a-5p, miR-342-3p and miR-195-5p in patients AT11 and AT12. Comparison between both blood cells samples (PBMCs and WBCs) **(B)**.
