# Supplementary figures and images for: MicroRNA dysregulation in ataxia telangiectasia
Source: Front Immunol. 2024 Aug 19;15:1444130. doi: 10.3389/fimmu.2024.1444130 (PMC11366618; doi:10.3389/fimmu.2024.1444130)

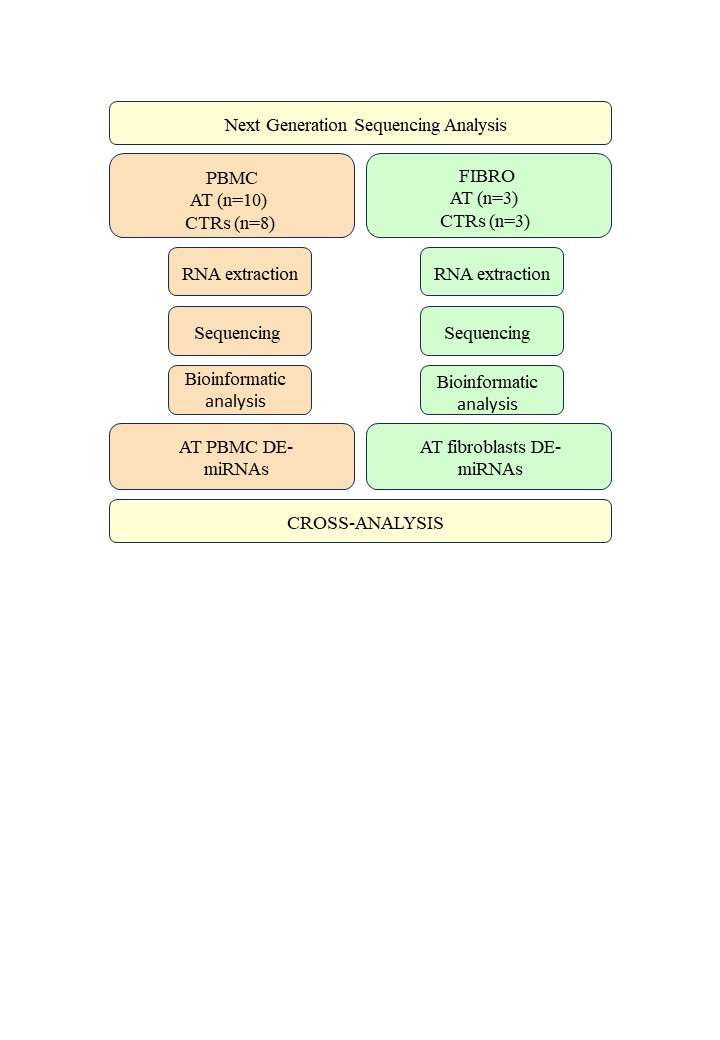

Supplement: Supplementary Figure 1 — Schematic workflow of the next generation sequencing analyses. The analysis was performed in PBMCs (orange) and fibroblasts (green) from AT patients and controls. [file Image1.jpeg]

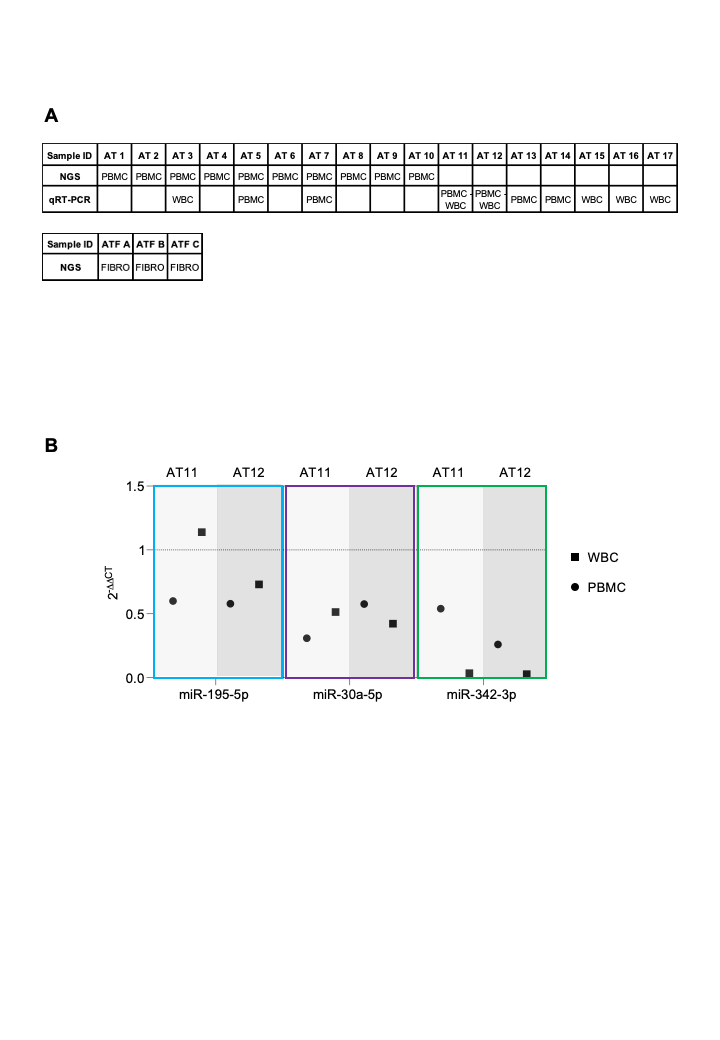

Supplement: Supplementary Figure 2 — AT blood cells and fibroblasts samples table. Patients were analyzed through NGS, qRT-PCR or both. Blood cells from AT patients were PBMCs, WBCs or both. NGS was performed on PBMC from AT1- AT10 patients and on fibroblasts of ATF A-C patients. qRT-PCR was performed on WBC (AT3), PBMC (AT5 and AT7) and on further 7 AT patients. Among these, the analysis was performed on both WBC and PBMC (AT1 and AT12), PBMC (AT13 and AT14) or WBC (AT15-AT17) (A). qRT-PCR of miR-30a-5p, miR-342-3p and miR-195-5p in patients AT11 and AT12. Comparison between both blood cells samples (PBMCs and WBCs) (B). [file Image2.tiff]
