# Supplementary material for: MicroRNA dysregulation in ataxia telangiectasia
Source: Front Immunol. 2024 Aug 19;15:1444130. doi: 10.3389/fimmu.2024.1444130 (PMC11366618; doi:10.3389/fimmu.2024.1444130)
Supplement: Supplementary file 3 [file Table1.docx]

**Table S1.** Genotype of AT cohort

| Patient ID | Genomic mutation  (Allel 1) | Phenotipic Effects  (Allel 1) | Molecular consequence | Genomic mutation (Allel 2) | Phenotipic Effects  (Allel 2) | Molecular consequence | Genotype |
| --- | --- | --- | --- | --- | --- | --- | --- |
| AT1 | c.3576G>A | p.? | Splicing | c.3576G>A | p.? | Splicing | Hom |
| AT2 | c.3526_3535del10 | p.(Leu1176_Ser1179Leufs) | Frameshift | c.3526_3535del10 | p.Leu1176_Ser1179>LeufsX1 | Frameshift | Hom |
| AT3 | c.6679C>T | p.(Arg2227Cys) | Missense | c.6679C>T | p.Arg2227Cys | Missense | Hom |
| AT4 | c.3894-3895insT | p.(Ala1299fs) | Frameshift | c.3894-3895insT | p.(Ala1299fs) | Frameshift | Hom |
| AT5 | c. 7517.7520delGAGA | p.(Arg2506fs) | Frameshift | c.7517_7520delGAGA | p.(Arg2506Thrfs) | Frameshift | Hom |
| AT6 | c.3576G>A | p.? | Splicing | c.3576G>A | p.? | Splicing | Hom |
| AT7 | c.8629insC | p.(Leu2877Serfs*2) | Frameshift | c.8977C>T | p.(Arg2993*) | Nonsense | Het |
| AT8 | c.8977C>T | p.(Arg2993*) | Nonsense | c.3802delG | p.(Val1268fs*) | Frameshift | Het |
| AT9 | c.5975_5979delAAAGT | p.(Lys1992fs) | Frameshift | c.9170G>C | p.(Ter3057Ser) | Missense | Het |
| AT10 | c.5975_5979delAAAGT | p.(Lys1992fs) | Frameshift | c.9170G>C | p.(Ter3057Ser) | Missense | Het |
| AT11 | c.381delA | p.(Val128*) | Frameshift | c.6679C>T | p.(Arg2227Cys) | Missense | Het |
| AT12 | c.97delC | p.(Arg33Glyfs) | Deletion | c.2113delT | p.(Tyr705Thrfs) | Frameshift | Het |
| AT13 | c.67C>T | p.(Arg23*) | Nonsense | c.3576G>A | p.? | Splicing | Het |
| AT14 | c.7517_7520delGAGA | p.(Arg2506Thrfs) | Frameshift | c.7408T>G | p.(Tyr2470Asp) | Missense | Het |
| AT15 | c.2413C>T | p.(Arg805*) | Nonsense | c.6995_6998del | p.(Leu2332fs) | Frameshift | Het |
| AT16 | c.6326G>A | p.(Trp2109*) | Nonsense | c.7408T>G | p.(Tyr2470Asp) | Missense | Het |
| AT17 | c.3894-3895insT | p.(Ala1299fs) | Frameshift | c.3894-3895insT | p.(Ala1299fs) | Frameshift | Het |
| ATF A | c.331+2T>G | p.? | Splicing | c.30_2816dup41kb | Exons 4-20 dup | Splicing | Het |
| ATF B | c.1607+1G>T | p.(Cys536fs) | Splicing | c.3576G>A | p.? | Splicing | Het |
| ATF C | c.1369C>T | p.(Arg457*) | Nonsense | c.3576G>A | p.? | Splicing | Het |

*Hom*, homozygous; *He*t, compound heterozygous.
